# Improved muscle and fat segmentation for body composition measures on quantitative CT

**DOI:** 10.1007/s11548-025-03466-2

**Published:** 2025-07-01

**Authors:** Jianfei Liu, Praveen Thoppey Srinivasan Balamuralikrishna, Sovira Tan, Pritam Mukherjee, Tejas Sudharshan Mathai, Perry J. Pickhardt, Ronald M. Summers

**Affiliations:** 1 Imaging Biomarkers and Computer-Aided Diagnosis Laboratory, Clinical Center, National Institutes of Health, 10 Center Dr, Bethesda, MD 20892 USA; 2 National Institute of Arthritis and Musculoskeletal and Skin Diseases, National Institutes of Health, 1 AMS Circle, Bethesda, MD 20892 USA; 3 Department of Radiology, University of Wisconsin School of Medicine and Public Health, 750 Highland Ave, Madison, WI 53726 USA

**Keywords:** Body composition, nnU-Net, Bone densitometry phantom, Muscle and fat segmentation

## Abstract

**Purpose:**

Body composition analysis on abdominal CT scans is useful for opportunistic screening. It also offers prognostic insights into mortality and cardiovascular risk. However, current segmentation methods for muscle and fat often fail on quantitative CT scans used for bone densitometry. These scans are commonly used to diagnose and monitor osteoporosis. This study aims to develop an accurate segmentation method for such scans and compare its performance with existing methods.

**Methods:**

We applied an nnU-Net framework to segment muscle, subcutaneous fat, visceral fat, and an added ‘body’ class for other non-background voxels. Training data included CT scans with bone densitometry phantoms, with segmentation annotations generated using our previous segmentation method followed by manual refinement. The proposed method was evaluated on 980 CT scans across two internal and external datasets, including 30 CT scans with phantoms in internal and external datasets (15 scans in each). Comparison was made with TotalSegmentator and our previous approach.

**Results:**

The proposed method achieved the highest accuracy for muscle and subcutaneous fat segmentation across all four datasets ($p<0.05$) and delivered comparable accuracy for visceral fat. In comparison with TotalSegmentator and the previous method, there were no false segmentations in the densitometry phantom included within the display field-of-view of the patient scan.

**Conclusion:**

Experimental results showed that the proposed method improved segmentation accuracy for muscle and subcutaneous fat while maintaining high accuracy for visceral fat. Notably, segmentation accuracy was also high in the quantitative CT scans for bone densitometry. These findings highlight the potential of the method to advance body composition analysis in clinical practice.

## Introduction

Automated body composition assessment on CT scans using artificial intelligence provides valuable insights into prognostic markers such as frailty, cardiovascular risks, and cancer cachexia [1]. It also offers the ability to predict mortality in patients with cirrhosis [2]. Recent work has shown that AI-driven CT body composition measure can serve as a valuable prognostic tool, enhancing the transition from opportunistic screening to broader clinical implementation [3].

Body composition assessment requires accurate volumetry of skeletal muscle and abdominal fat [1–3], driving the improvement of automated segmentation methods [4–16]. These methods are generally categorized as slice-based or scan-based. Slice-based approaches analyze CT slices at the L1 or L3 vertebral levels. For example, a set of manually annotated CT slices at the L3 level was used to train 15 2D U-Net models, that were then ensembled for muscle segmentation [4]. The identification of the L3 level was automated using a YOLOv3-based algorithm [5].

In our earlier work, a dual-branch segmentation model was developed to generate annotations for unannotated CT slices using a small number of annotated examples [8]. This method facilitated scan-based segmentation by providing training data for 3D U-Net models with higher segmentation accuracy [6, 9]. A 3D multi-resolution U-Net was also employed to segment muscle and fat, with improved accuracy achieved by refining Hounsfield Unit limits [17]. Similar thresholding strategies have been proposed to separate soft tissue, bone, and air [7], or to enhance visceral fat segmentation using a Gaussian kernel density function [15]. U-Net was also used to segment the ventral cavity, followed by threshold-based methods to segment visceral and subcutaneous fat [18]. The inclusion of additional segmentation classes, such as skin, internal organs with vessels, and the central nervous system, further enhanced segmentation performance [11]. TotalSegmentator (TS) [12] incorporated over 100 segmentation classes and was built on the state-of-the-art nnU-Net framework [13]. The self-configuration capability of this framework allowed automatic optimization of training parameters and dataset adaptation. In our recent work, an nnU-Net-based muscle and fat segmentation method [14] was developed using training data efficiently generated by a dual-branch network [8]. A comparative study with TS as the baseline demonstrated the state-of-art segmentation accuracy of this approach.

**Fig. 1:**
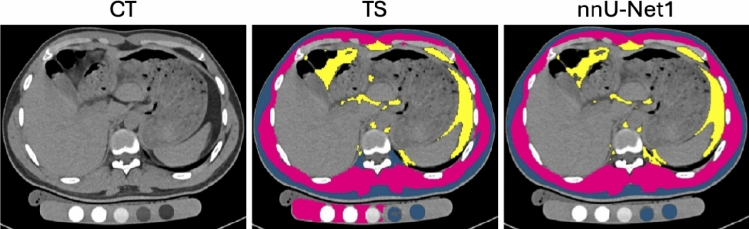
Comparison of muscle and fat segmentation on a quantitative CT scan. Blue represents subcutaneous fat, pink indicates muscle, and yellow denotes visceral fat. TotalSegmentator (TS) exhibits false muscle segmentation in the bone densitometry phantom located beneath the patient, while both TS and our previous segmentation method (nnU-Net1) show false subcutaneous fat segmentation in the phantom

However, these methods often fail on quantitative CT scans for bone densitometry [19] in which false positives occur in the bone densitometry phantom (Fig. 1). Quantitative CT is routinely used to assess bone mineral density for diagnosing and monitoring osteoporosis [17]. Errors in muscle and fat segmentation in bone densitometry phantoms can lead to inaccuracies in body composition measurements. To the best of our knowledge, only one study has assessed automatic segmentations of regional muscle and intermuscular fat on quantitative CT and it focused on the slice-based approach [20]. This limitation motivated us to improve our method to generate accurate muscle and fat segmentation on quantitative CT scans.

## Methods

### Data collection

The dataset consisted of 1090 CT scans from three internal and two external datasets. Of these, 40 were quantitative CT scans, and the remainder were abdominal CT scans. Table 1 summarizes the patient demographics for all five datasets. The first internal dataset (IN1) was for a study on patients with sarcopenia [21]. It was used to train our previous segmentation methods [8, 14]. It was also utilized to train the improved method in this work. The second internal dataset (IN2) comprised 25 quantitative CT scans from 25 patients. Ten scans were used as additional training data and the remaining 15 served as the testing dataset. The third internal dataset (IN3) includes 50 abdominal CT scans from 50 patients with chronic granulomatous disease [22]. They were used to evaluate segmentation performance on routine abdominal CT scans. All three internal datasets were acquired at the National Institutes of Health Clinical Center. The first external dataset (EXT1) consists of 900 abdominal CT scans obtained from various institutions through The Cancer Imaging Archive (TCIA) [23]. The second external dataset (EXT2) includes 15 quantitative CT scans randomly selected from follow-up CT scans within a large data archive. This archive was specifically designed for patients undergoing CT colonography studies at the University of Wisconsin (UW) [24]. The collected data included both contrast-enhanced and non-contrast CT scans, encompassing a wide range of patient ages and originating from multiple institutions. This diversity provided a robust foundation for training and testing the enhanced segmentation method.

**Table 1:** Patient demographics of three internal (IN) and two external datasets (EXT)

Dataset	IN1	IN2	IN3	EXT1	EXT2
Number (n)	100	25	50	900	15
Age (years)	$66.7 \pm 6.3$	$45.0 \pm 12.9$	$20.3 \pm 11.3$	$57.7 \pm 5.8$	$62.8 \pm 11.4$
	(59–87)	(22–68)	(4–45)	(20–85)	(51–87)
Men (n)	56	21	38	–	5
Women (n)	44	4	12	–	10
IV contrast present (n)	98	0	30	589	0
IV contrast absent (n)	2	25	20	321	15
Data source	NIH	NIH	NIH	TCIA	UW

For training and validation, all abdominal CT scans in the IN1 dataset were annotated by combining segmented CT slices generated by the dual-branch network [8] with a small number of manually annotated slices. For the IN2 dataset, subcutaneous fat, visceral fat, and muscle were automatically segmented on 10 quantitative CT scans using our previous nnU-Net1 method. False segmentations in the bone densitometry phantom were then manually corrected by a staff scientist. These 10 quantitative CT scans, combined with 100 abdominal CT scans from IN1, formed the data for training and validating the enhanced method. The 110 scans were evenly divided into five folds. A five-fold cross-validation strategy was applied, using four folds for training and one fold for validation in each split. This process resulted in five enhanced segmentation models, as described below. Each model was trained on approximately 88 scans and validated on 22 scans.

For testing, we employed two internal datasets (IN2 and IN3) and two external datasets (EXT1 and EXT2). The remaining 15 quantitative CT scans in IN2 were manually annotated by Radiology Resident 1. Five slices per scan were randomly selected to cover the range from chest to pelvis, and subcutaneous fat, visceral fat, and muscle were annotated. The same slice selection strategy was employed by Radiology Resident 2 for annotating CT slices in the IN3 dataset. Resident 1 also provided sparse annotations across all 15 quantitative CT scans in the EXT2 dataset. For the EXT1 dataset, a set of CT slices was manually annotated across all abdominal CT scans, with an interval of 5 mm between annotated slices, though visceral fat was not annotated. All CT scans with sparse slice annotations across the datasets were used as the testing dataset for the enhanced segmentation method. Only the annotated slices in the testing dataset were used to compare segmentation results. Unannotated slices were excluded from evaluation. Additionally, CT scans with sparse slice annotations were not used for training.

TS [12] and nnU-Net1 [14] were chosen as the baseline methods. nnU-Net1 was trained on the IN1 dataset, while the proposed nnU-Net2 used both the IN1 dataset and 10 additional quantitative CT scans from the IN2 dataset. The Dice Similarity Coefficient (DSC), Hausdorff Distance (HD), and Mean Absolute Difference (MAD) were used to evaluate segmentation accuracy. Higher DSC values indicate better agreement between the predicted and ground truth masks. In contrast, lower HD and MAD values reflect more accurate and consistent segmentation results.

### Improved muscle and fat segmentation method

The improved segmentation method was developed using the latest nnU-Net v2 framework. It offers superior performance and reduced inference time in comparison with our previous method trained on the nnU-Net v1 framework [14]. The segmentation mask of our previous nnU-Net1 method included four classes: background, subcutaneous fat, visceral fat, and muscle. This work introduces an additional ‘body’ class to address the significant heterogeneity within the broad background class of nnU-Net1. The ‘body’ class contains all object voxels excluding those annotated as muscle or fat. To achieve this, TS was utilized to segment body regions in all CT scans within the training dataset. Minor manual edits were made to exclude any extraneous body segmentations at the CT bed or bone densitometry phantoms. The subcutaneous fat, visceral fat, and muscle annotations were then assigned distinct annotations within the refined body segmentation mask. This resulted in the final segmentation mask for the improved method.

The default nnU-Net2 settings were applied: 1000 epochs, a combinatorial loss of categorical (multi-class) cross-entropy and multi-class soft Dice, the SGD optimizer with an initial learning rate of $10^{-3}$, and a batch size of 1. Let *C* be the number of classes and *N* be the number of voxels in a CT scan. In this work, $C=3$ corresponds to subcutaneous fat, visceral fat, and muscle. Let $y_{i,c}\in \{0,1\}$ and $\hat{y}_{i,c}\in [0,1]$ represent the one-hot encoded ground truth and the predicted probability for voxel *i*, class *c*, respectively. The categorical cross-entropy loss (CCE) is defined as:

1 $$\begin{aligned} L_{CCE} =-\frac{1}{N}\sum _{i=1}^N\sum _{c=1}^Cy_{i,c}\log (\hat{y}_{i,c}) \end{aligned}$$

This loss measures the voxel-wise classification error across all classes, including of subcutaneous fat, visceral fat, and muscle.

The multi-class Dice loss is defined as

2 $$\begin{aligned} L_{Dice} =1-\frac{1}{C}\sum _{c=1}^C\hbox {Dice}_{c} \end{aligned}$$

where

3 $$\begin{aligned} \hbox {Dice}_{c} =\frac{2\sum _{i=1}^Ny_{i,c}\hat{y}_{i,c} + \epsilon }{\sum _{i=1}^Ny_{i,c}+\sum _{i=1}^N\hat{y}_{i,c} + \epsilon } \end{aligned}$$

The term $\epsilon$ is a small constant added for numerical stability. Combining Eqs. 1 and 2 leads to the combinatorial loss.

4 $$\begin{aligned} L_{Combined}=L_{CCE}+L_{Dice} \end{aligned}$$

Five muscle and fat segmentation models with different initializations were trained using the five-fold split described in the previous section. The final segmentation results were obtained by ensembling the outputs from these five models. All segmentation models were trained on an A100 40GB SXM GPU card. All validation experiments were also performed on the same GPU card.

## Experimental results

**Fig. 2:**
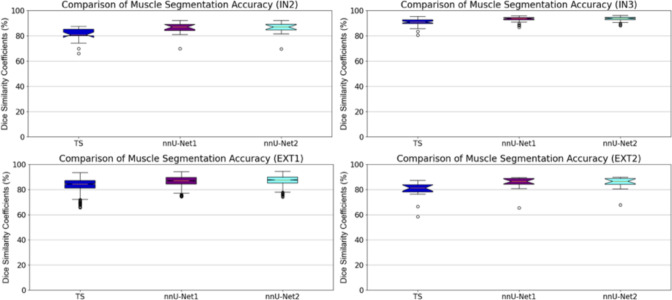
Comparison of muscle segmentation results using TS, nnU-Net1 and nnU-Net2 on two internal (top row) and two external (bottom row) datasets

**Fig. 3:**
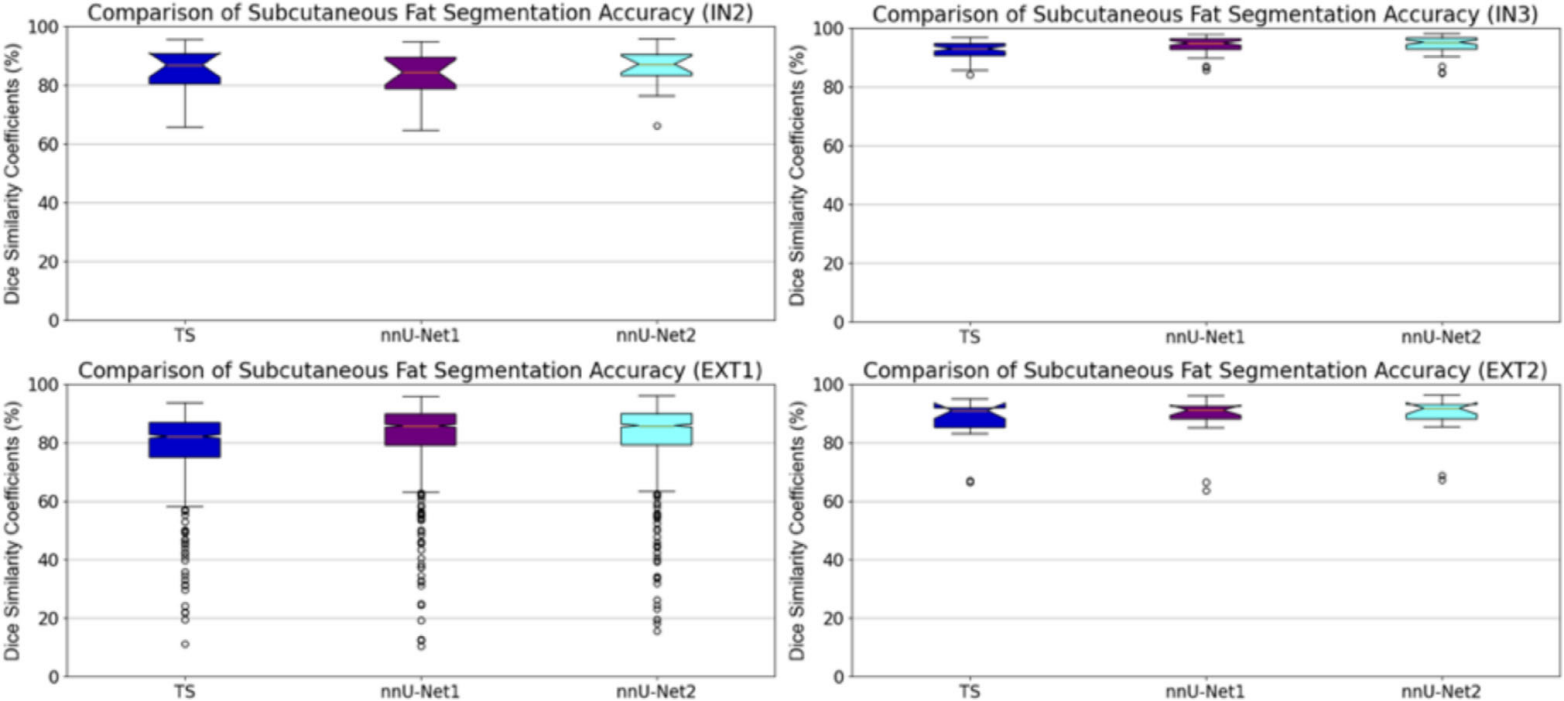
Comparison of subcutaneous fat segmentation results using TS, nnU-Net1 and nnU-Net2 on two internal (top row) and two external (bottom row) datasets

**Fig. 4:**
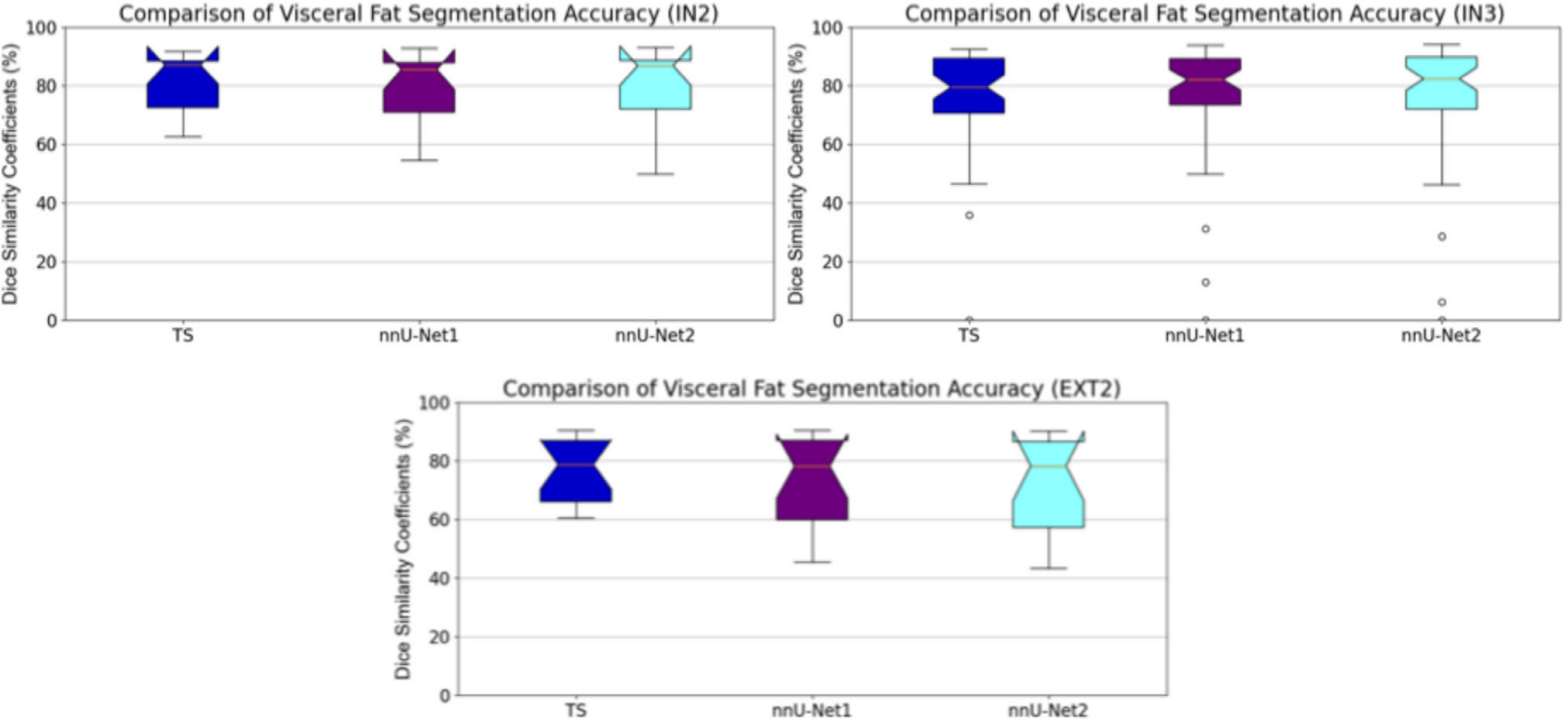
Comparison of visceral fat segmentation results using TS, nnU-Net1 and nnU-Net2 on two internal (top row) and two external (bottom row) datasets

Tables 2, 3, and 4 present a comparison of the segmentation results for muscle, subcutaneous fat, and visceral fat across two internal and two external datasets using DSC, HD, and MAD segmentation evaluation metrics. The improved segmentation method, nnU-Net2, achieved the highest DSC and MAD values for both muscle and subcutaneous fat segmentation. Notably, nnU-Net2 demonstrated statistically significant improvements in segmentation accuracy for muscle and subcutaneous fat compared to TS and nnU-Net1 ($p < 0.05$). In contrast, TS achieved higher DSC and HD values for visceral fat across all datasets. However, its results were not statistically significantly better than those of nnU-Net1 and nnU-Net2 ($p > 0.1$), except for the EXT2 dataset ($p < 0.05$). The number of CT scans in the EXT2 dataset may contribute to the observed significant differences in visceral fat segmentation accuracy. Figures 2, 3, and 4 provide boxplots illustrating the DSC values for muscle, subcutaneous fat, and visceral fat segmentation results.

**Table 2:** Comparison of muscle, subcutaneous fat, and visceral fat Dice similarity coefficient (DSC) values between TotalSegmentator (TS), our previous method (nnU-Net1), and the improved method (nnu-Net2) on two internal and two external datasets

Dataset	$\hbox {IN2}^*$	IN3	$\hbox {EXT1}^\dagger$	EXT2
Muscle				
TS	$80.5 \pm 6.1\%$	$90.8 \pm 2.7\%$	$82.8 \pm 4.6\%$	$79.5 \pm 7.5\%$
nnU-Net1	$85.7 \pm 5.4\%$	$93.2 \pm 2.1\%$	$86.8 \pm 3.7\%$	$85.1 \pm 6.0\%$
nnU-Net2	$\mathbf {86.0 \pm 5.4\%}$	$\mathbf {93.3 \pm 2.0\%}$	$\mathbf {87.4 \pm 3.5\%}$	$\mathbf {85.2 \pm 5.5\%}$
Subcutaneous fat				
TS	$85.4 \pm 8.0\%$	$92.6 \pm 2.9\%$	$79.8 \pm 10.6\%$	$86.9 \pm 8.5\%$
nnU-Net1	$84.0 \pm 8.4\%$	$94.2 \pm 2.8\%$	$82.7 \pm 11.3\%$	$87.8 \pm 9.3\%$
nnU-Net2	$\mathbf {86.2 \pm 7.4\%}$	$\mathbf {94.3 \pm 3.1\%}$	$\mathbf {82.8 \pm 11.1\%}$	$\mathbf {88.6 \pm 8.4\%}$
Visceral fat				
TS	$\mathbf {81.2 \pm 9.6\%}$	$78.0 \pm 13.5\%$	–	$\mathbf {76.7 \pm 11.1\%}$
nnU-Net1	$79.3 \pm 11.5\%$	$\mathbf {78.4 \pm 16.0\%}$	–	$73.2 \pm 15.3\%$
nnU-Net2	$79.4 \pm 12.9\%$	$78.0 \pm 17.8\%$	–	$72.5 \pm 15.8\%$

$^1$ $*$ Parts of quantitative CT (15) were used for evaluating segmentation accuracy. $^2$ $^{\dagger }$ Manual annotations of visceral fat were unavailable. $^3$ Bold numbers indicate the highest DSC values

**Table 3:** Comparison of muscle, subcutaneous fat, and visceral fat Hausdorff Distance (HD) values between TotalSegmentator (TS), our previous method (nnU-Net1), and the improved method (nnu-Net2) on two internal and two external datasets

Dataset	IN2	IN3	EXT1	EXT2
Muscle				
TS	$31.7 \pm 18.2$ mm	$5.8 \pm 2.1$ mm	$\mathbf {11.5 \pm 4.1}$ mm	$25.7 \pm 21.2$ mm
nnU-Net1	$20.3 \pm 20.4$ mm	$\mathbf {3.6 \pm 1.4}$ mm	$12.0 \pm 6.6$ mm	$15.4 \pm 18.7$ mm
nnU-Net2	$\mathbf {19.1 \pm 19.0}$ mm	$3.8 \pm 1.3$ mm	$11.8 \pm 7.3$ mm	$\mathbf {15.2 \pm 18.0}$ mm
Subcutaneous fat				
TS	$26.6 \pm 22.5$ mm	$9.8 \pm 5.4$ mm	$20.6 \pm 7.9$ mm	$15.4 \pm 6.7$ mm
nnU-Net1	$22.5 \pm 21.3$ mm	$5.1 \pm 7.0$ mm	$14.0 \pm 16.5$ mm	$10.0 \pm 8.6$ mm
nnU-Net2	$\mathbf {18.9 \pm 21.1}$ mm	$\mathbf {4.0 \pm 5.6}$ mm	$\mathbf {12.7 \pm 16.3}$ mm	$\mathbf {8.6 \pm 11.4}$ mm
Visceral fat				
TS	$\mathbf {10.3 \pm 5.0}$ mm	$\mathbf {12.1 \pm 12.6}$ mm	–	$\mathbf {16.6 \pm 9.3}$ mm
nnU-Net1	$20.0 \pm 13.9$ mm	$21.0 \pm 21.1$ mm	–	$19.4 \pm 12.6$ mm
nnU-Net2	$14.8 \pm 11.6$ mm	$21.1 \pm 21.5$ mm	–	$19.4 \pm 12.3$ mm

Bold numbers indicate the lowest HD values

**Table 4:** Comparison of muscle, subcutaneous fat, and visceral fat Mean Absolute Difference (MAD) values between TotalSegmentator (TS), our previous method (nnU-Net1), and the improved method (nnu-Net2) on two internal and two external datasets

Dataset	IN2	IN3	EXT1	EXT2
Muscle				
TS	$3.2 \pm 0.7\%$	$1.6 \pm 0.4\%$	$2.8 \pm 1.1\%$	$3.0 \pm 1.0\%$
nnU-Net1	$2.5 \pm 0.8\%$	$\mathbf {1.3 \pm 0.4\%}$	$2.3 \pm 1.0\%$	$\mathbf {2.4 \pm 0.8\%}$
nnU-Net2	$\mathbf {2.4 \pm 0.8\%}$	$\mathbf {1.3 \pm 0.4\%}$	$\mathbf {2.2 \pm 0.9\%}$	$\mathbf {2.4 \pm 0.8\%}$
Subcutaneous fat				
TS	$1.9 \pm 0.6\%$	$1.1 \pm 0.4\%$	$3.3 \pm 1.1\%$	$2.1 \pm 0.7\%$
nnU-Net1	$2.0 \pm 0.6\%$	$\mathbf {0.8 \pm 0.3\%}$	$2.5 \pm 0.7\%$	$1.8 \pm 0.5\%$
nnU-Net2	$\mathbf {1.7 \pm 0.6\%}$	$\mathbf {0.8 \pm 0.3\%}$	$\mathbf {2.5 \pm 0.6\%}$	$\mathbf {1.6 \pm 0.5\%}$
Visceral fat				
TS	$\mathbf {1.4 \pm 0.7\%}$	$1.6 \pm 0.4\%$	–	$\mathbf {1.7 \pm 0.4\%}$
nnU-Net1	$1.5 \pm 0.8\%$	$\mathbf {1.3 \pm 0.4\%}$	–	$1.8 \pm 0.4\%$
nnU-Net2	$\mathbf {1.4 \pm 0.7\%}$	$\mathbf {1.3 \pm 0.4\%}$	–	$\mathbf {1.7 \pm 0.4\%}$

Bold numbers indicate the lowest MAD values

**Fig. 5:**
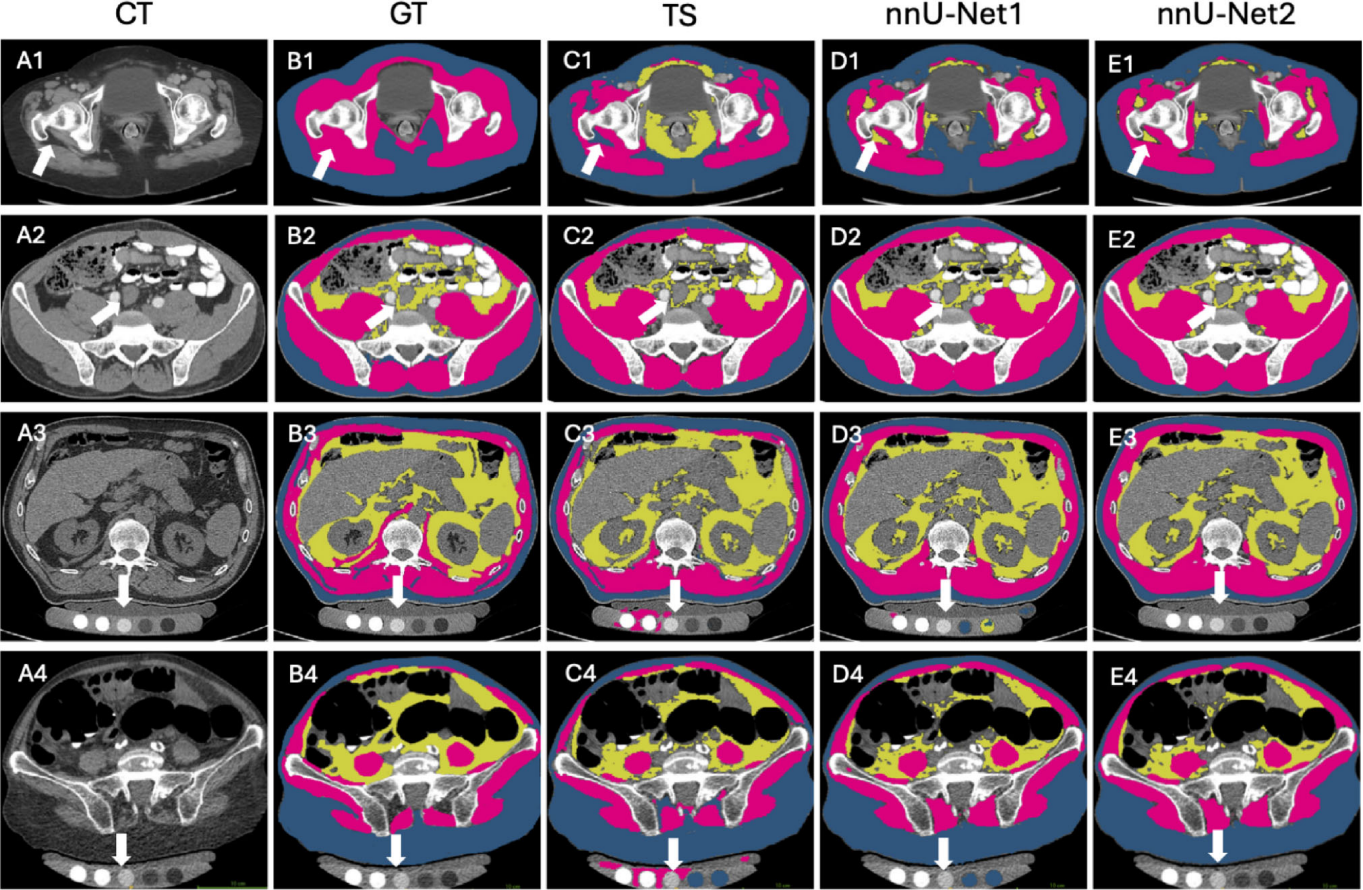
Example segmentation results from four datasets: EXT1 (A1-E1), IN3 (A2-E2), IN2 (A3-E3), and EXT2 (A4-E4), using the TS, nnU-Net1, and nnU-Net2 methods

Figure 5 presents four example segmentation results selected from the testing datasets listed in Table 1. These examples include abdominal CT scans from EXT1 and IN3 (top two rows) and quantitative CT scans from IN2 and EXT2 (bottom two rows). In the abdominal CT scans (A1–E1, A2–E2), TS, nnU-Net1, and nnU-Net2 effectively segmented subcutaneous fat and muscle. However, some disagreements were observed in visceral fat segmentation. In the ground truth (GT, B1), intra-muscular fat was annotated as muscle (white arrow). However, this region was not captured in the TS results (C1) and was segmented as visceral fat by nnU-Net1 and nnU-Net2. In the other example, the TS results for visceral fat (C2) showed slight under-segmentation, whereas nnU-Net1 and nnU-Net2 provided more accurate segmentation. For the quantitative CT scans, nnU-Net2 achieved accurate segmentation of subcutaneous fat, visceral fat, and muscle in both the internal (A3–E3) and external datasets (A4–E4). In contrast, TS and nnU-Net1 displayed false segmentations of subcutaneous fat, visceral fat, and muscle, particularly in regions containing bone densitometry phantoms.

**Fig. 6:**
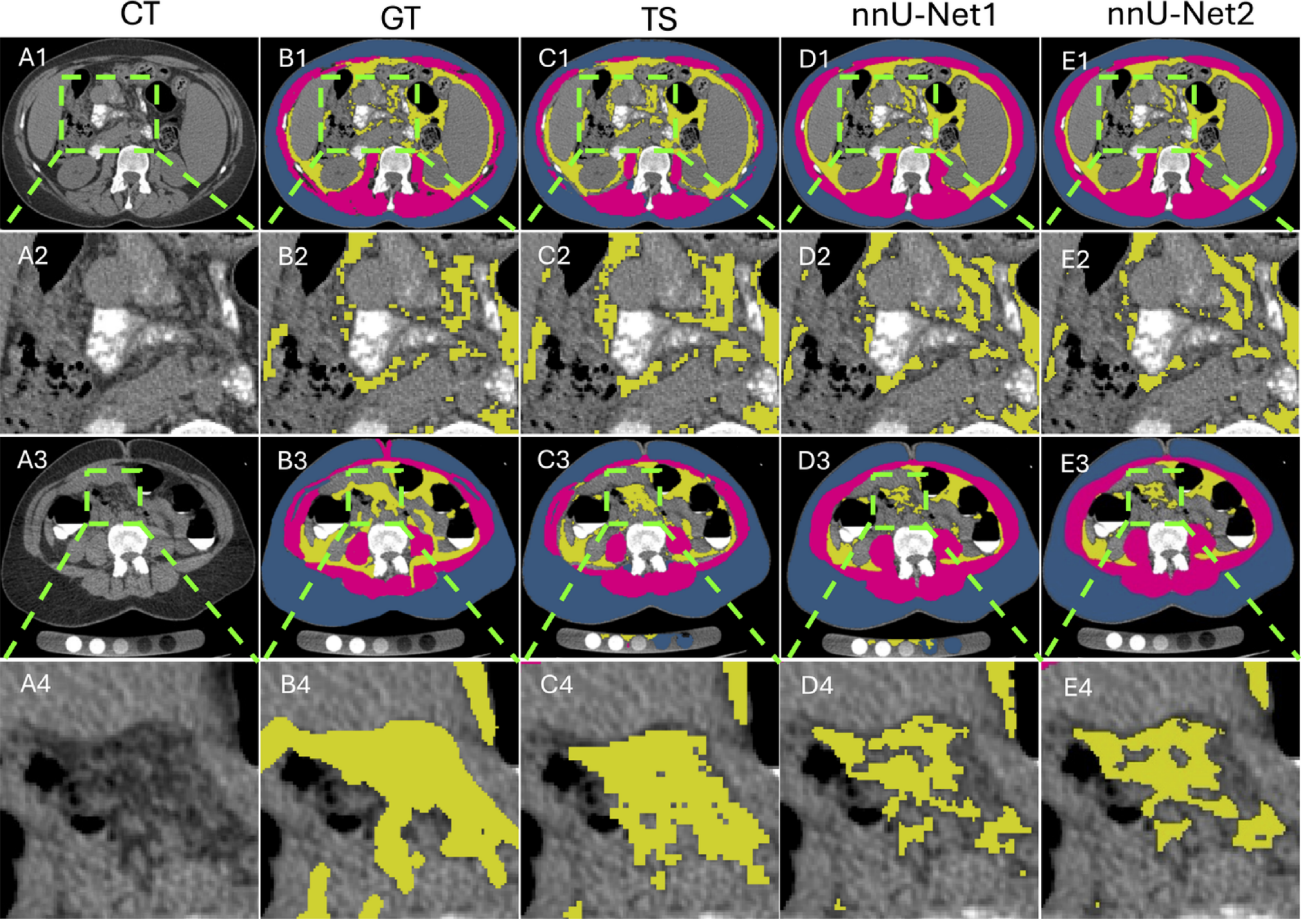
Segmentation results with errors (indicated by white arrows) from two datasets, IN3 (A1–E1) and EX2 (A3–E4), using the TS, nnU-Net1, and nnU-Net2 methods. Panels A2–E2 and A4–E4 show the corresponding zoomed-in views, highlighting the challenges of visceral fat segmentation caused by its spotted and irregular distribution

One common segmentation error observed in our experimental results (Fig. 6) was the under-segmentation of visceral fat in the abdominal and pelvic regions. This challenge arises from the inherently spotted and irregular distribution of visceral fat, as illustrated in the zoomed-in views (A2–E2 and A4–E4).

## Conclusion and future work

In this paper, we developed an improved muscle and fat segmentation method based on the nnU-Net V2 framework to address segmentation errors in quantitative CT scans. State-of-the-art segmentation methods, including TotalSegmentator and our previous approach, exhibited false segmentation in regions containing bone densitometry phantoms, leading to incorrect muscle and fat segmentation. Such errors can affect the calculation of body composition metrics, which rely on accurate muscle and fat volume measurements.

To resolve this issue, we incorporated quantitative CT scans into the training data and introduced an additional ‘body’ class. The ‘body’ class reduces heterogeneity outside the annotated muscle and fat regions. The improved segmentation method not only addressed the issue of phantom-induced errors but also enhanced the segmentation accuracy for muscle and subcutaneous fat. In terms of computational efficiency, TotalSegmentator achieved the lowest average processing time on the IN1 dataset, with approximately 18.2 s per scan. In comparison, nnU-Net1 and nnU-Net2 required 25.2 and 25.1 s per scan, respectively. This observation aligns with the original statement in the nnU-Net development paper, which noted minimal differences in computational efficiency between nnU-Net1 and nnU-Net2 [13]. To the best of our knowledge, we are the first to present a 3D segmentation approach to improve muscle and fat segmentation accuracy for body composition in quantitative CT scans.

The proposed nnU-Net2 produced lower DSC values for visceral fat compared to both TotalSegmentator and nnU-Net1. The spotted and irregular distribution of visceral fat (Fig. 6) significantly increases the difficulty of manual annotation. Radiology residents often annotated broad mixed regions that include not only fat but also adjacent small vessels and soft tissues, potentially leading to over-segmentation of the visceral fat region (B1-B4, Fig. 6). TotalSegmentator may retain these non-fat components, possibly due to being trained on labels that include such over-segmented regions (C1-C4). In contrast, nnU-Net1 and nnU-Net2 were trained using thresholding-based labels constrained to the fat intensity range, followed by manual refinement. This approach, while more specific to fat, may exclude surrounding structures and result in lower DSC when compared to models trained on broader annotations (D1-D4, E1-E4). Nevertheless, all methods achieved comparable DSC values in visceral fat segmentation. Future work will focus on updating the ground truth annotations to further improve consistency and reliability. One limitation of this study is that only a small number of slices in each CT scan were manually annotated. This sparse annotation strategy is commonly adopted in publicly available datasets, such as SAROS [23] and AATCT-IDS [25]. In this work, the SAROS dataset (EXT1) was used to validate the performance of the proposed nnU-Net2 method. Similar sparse annotation approaches have been employed in prior studies to train and evaluate muscle and fat segmentation models [2–6, 10, 15, 16, 20], aligning with our methodology. The CT slice at the L3 vertebral level holds particular clinical significance, as it is widely used for assessing body composition in various contexts, including predicting outcomes in patients with cirrhosis [2], evaluating sarcopenia [4], and serving as a prognostic tool for oncologic patients in opportunistic screening [3]. Automated methods have also been developed to select the L3 slice to facilitate body composition analysis on CT scans [5]. There are only a limited number of studies that annotate entire CT scans, due to the highly time-consuming nature of the task [7]. To reduce annotation burden, this study contoured only the abdominal muscular wall and subsequently identified fat regions using intensity thresholding. However, even with this simplified strategy, annotating visceral fat on CT scans with a 4mm slice thickness still required over 30 minutes per scan. Moreover, threshold-based segmentation without manual refinement can lead to inaccurate results. For these reasons, this work followed the mainstream practice of using sparse slice annotation to evaluate the segmentation accuracy of muscle and fat. Future work will focus on developing efficient and accurate methods for annotating muscle and fat across the entire CT scan, with particular emphasis on visceral fat. Furthermore, only 30 quantitative CT scans were used in this study to validate segmentation accuracy. This limited number of annotated test cases may have underestimated the performance gains achieved by the proposed nnU-Net2 method. Future efforts will focus on collecting a larger database of quantitative CT scans to further evaluate the impact of enhanced segmentation accuracy on body composition assessment.

In conclusion, we developed an enhanced segmentation method to accurately segment muscle and adipose tissue, enabling reliable body composition assessment on quantitative CT scans. This improved assessment of fat and muscle distribution is clinically valuable for evaluating fracture risk and bone mineral density, as highlighted in studies on Chinese and Korean populations [26, 27]. It is particularly useful for detecting osteoporosis risk in normal-weight individuals, where conventional indicators may be less reliable [28].

## Data Availability

Code will be made available on request.
